# Supplementary material for: Biophysical models reveal the relative importance of transporter proteins and impermeant anions in chloride homeostasis
Source: eLife. 2018 Sep 27;7:e39575. doi: 10.7554/eLife.39575 (PMC6200395; doi:10.7554/eLife.39575)
Supplement: Supplementary file 1. [file elife-39575-supp1.pdf]

# Analytical solution derivation

We wanted to find analytical solutions at steady state for the following variables in the pump-leak formulation for a single compartment including KCC2: intracellular concentrations of sodium, potassium, chloride and impermeant anions ($[Na^+]_i$; $[K^+]_i$; $[Cl^-]_i$; and $[X^z]_i$, with charge $z$); and membrane voltage ($V$). The steady state should occur in the presence of both a pump leak mechanism (sodium-potassium ATPase with pump rate modified by the sodium gradient, $J_p = p \cdot \left(\frac{[Na^+]_i}{[Na^+]_o}\right)^3$) and chloride-potassium extrusion (type 2 potassium-chloride co-transporter, KCC2, with conductance $g_{KCC2}$ and driving force proportional to the difference in the Nernst reversal potentials of potassium and chloride — see Doyon et al. (2016)). The usual passive forces acting across the membrane on each ion are also included. To allow for an analytical solution inclusive of differences in osmolarity between the intracellular and extracellular environments (**Fig. 6A-E**), we derive the analytical solution with $\Pi_i = \Pi_o + N_{H_p}$, where $\Pi_i$ is the intracellular osmolarity and $\Pi_o$ the extracellular. Thus $N_{H_p}$ is often but not always equal to 0.

The situation described above ought to satisfy the following five equations at steady state, in which the conductance of an ion is denoted $g_{ion}$, an ion's Nernst reversal potential as $E_{ion}$, $F$ the Faraday constant and $A_m$ the ratio of surface area to volume. This system is similar to that given in Keener and Sneyd (2009).

$$-\frac{d}{dt}\left(\frac{F}{A_m}[Na^+]_i\right) = g_{Na}(V - E_{Na}) + 3J_p \tag{S1}$$

$$-\frac{d}{dt}\left(\frac{F}{A_m}[K^+]_i\right) = g_K(V - E_K) - 2J_p - g_{KCC2}(E_K - E_{Cl}) \tag{S2}$$

$$\frac{d}{dt}\left(\frac{F}{A_m}[Cl^-]_i\right) = g_{Cl}(V - E_{Cl}) + g_{KCC2}(E_K - E_{Cl}) \tag{S3}$$

$$0 = [K^+]_i + [Na^+]_i - [Cl^-]_i + z[X^z]_i \tag{S4}$$

$$\Pi_o + N_{H_p} = \Pi_i = [K^+]_i + [Na^+]_i + [Cl^-]_i + [X^z]_i \tag{S5}$$

We first solve the system for constant $J_p$ at steady state, i.e. equations S1, S2 and S3 set to 0, and then show that a parametric solution exists for $p$ such that the function mapping $J_p$ to $p$ is bijective. Thus we begin by solving each of (S1), (S2) and (S3) for the reversal potential of the intracellular ion that they refer to, and then for the intracellular ions' concentration itself. By simple rearrangement,

$$[Na^+]_i = [Na^+]_o \cdot e^{-\frac{FV}{RT}} \cdot e^{-\frac{3J_pF}{RTg_{Na}}} \tag{S6}$$

and

$$E_{Cl} = \frac{g_{Cl}V + g_{KCC2}E_K}{g_{Cl} + g_{KCC2}} \tag{S7}$$

Let $\beta$ be equal to $g_K g_{Cl} - g_{KCC2} g_{Cl} + g_K g_{KCC2}$. If we substitute (S7) into (S2) for $E_{Cl}$, we can solve for $E_K$ and $[K^+]_i$, hence enabling us to substitute back into (S7) in order to solve for $[Cl^-]_i$.

Thus,

$$E_K = V - 2J_p \frac{g_{Cl} + g_{KCC2}}{\beta}$$

and hence

$$[K^+]_i = [K^+]_o \cdot e^{-\frac{FV}{RT}} \cdot e^{\frac{F}{RT} \cdot 2J_p \frac{g_{Cl}+g_{KCC2}}{\beta}} \tag{S8}$$

so then

$$[Cl^-]_i = [Cl^+]_e \cdot e^{\frac{FV}{RT}} \cdot e^{-\frac{F}{RT} \cdot \frac{2J_p \cdot g_{KCC2}}{\beta}} \tag{S9}$$

We have now found equations for all permeant intracellular ions in terms of constants and $V$. An extension of these results means that we can find an equation for $X$ in terms of $V$ by rearranging the osmotic equilibrium equation (S5).

$$[X^z]_i = (\Pi_o + N_{H_p}) - [Na^+]_i - [K^+]_i - [Cl^-]_i \tag{S10}$$

In order to solve for $V$, we substitute (S10) into (S4), the equation that ensures intracellular charge neutrality. Thus we obtain:

$$0 = z \cdot (\Pi_o + N_{H_p}) + (1 - z) \cdot ([K^+]_i + [Na^+]_i) - (1 + z) \cdot [Cl^-]_i \tag{S11}$$

Before substituting in for the permeable intracellular ions, let us denote $\theta = e^{-\frac{FV}{RT}}$. Then with substitution of (S9), (S8) and (S6) and multiplication through by $\theta$ equation S11 becomes:

$$0 = (1-z) \cdot \left([K^+]_o \cdot e^{\frac{2J_pF \cdot (g_{Cl}+g_{KCC2})}{RT \cdot \beta}} + [Na^+]_o \cdot e^{-\frac{3J_pF}{RT \cdot g_{Na}}}\right) \cdot \theta^2 + z \cdot (\Pi_o + N_{H_p}) \cdot \theta - (1+z) \cdot [Cl^-]_o \cdot e^{\frac{-2J_pF \cdot g_{KCC2}}{RT \cdot \beta}}$$

This quadratic equation can be solved in terms of $\theta$ using the quadratic formula.

$$\theta = \frac{-z \cdot (\Pi_o + N_{H_p}) + \sqrt{z^2 \cdot (\Pi_o + N_{H_p})^2 + 4(1 - z^2) \cdot [Cl^-]_o \cdot e^{-\frac{2J_pF \cdot g_{KCC2}}{RT \cdot \beta}} \cdot \left([Na^+]_o \cdot e^{-\frac{3J_pF}{RT \cdot g_{Na}}} + K_e \cdot e^{\frac{2J_pF \cdot (g_{Cl}+g_{KCC2})}{RT \cdot \beta}}\right)}}{2 \cdot (1 - z) \cdot \left([Na^+]_o \cdot e^{-\frac{3J_pF}{RT \cdot g_{Na}}} + [K^+]_o \cdot e^{\frac{2J_pF \cdot (g_{Cl}+g_{KCC2})}{RT \cdot \beta}}\right)} \tag{S12}$$

From this one can solve the system for any constants — at least those constants which give positive real solutions for $\theta$ — and then use $V = -\frac{RT}{F} \ln \theta$ to transform the solution into the corresponding membrane voltage. This implies that initial values of the intracellular ion concentrations do not affect the final steady state (this includes shifts in $X$ that do not change the average intracellular charge $z$). Note that (S12) when $z = -1$, to avoid division by 0, the solution is found by substituting $z = -1$ into (S11) and simplifying.

Finally, we extend the solution from the constant pump rate assumed above to a pump rate modulated by the sodium concentration, as used in our model. The sodium-dependent pump rate updated by $J_p = p \cdot \left(\frac{[Na^+]_i}{[Na^+]_o}\right)^3$ cannot be solved purely analytically because one ends up attempting to find a solution for an expression unsolvable in the reals (*W-Lambert Function*). In this case, one might substitute different values of $J_p$ into the solution above, and then use the function $f(J_p, [Na^+]_i)$ defined by $J_p = p \cdot \left(\frac{[Na^+]_i}{[Na^+]_o}\right)^3$ to solve for $p$.

$f$ rearranged with $p$ as the subject of the formula resembles a parametric function. Were $p$ an independent variable determining the ionic solutions of the analytic solution, each simulation beginning with constant $p$ would have a unique steady

state. To make this claim, the function mapping $J_p$ and the steady state $[Na^+]_i$ to $p$ needs to be injective (more strictly, $f : J_p \rightarrow p$ must be bijective). The reason for this constraint is that if ever a $p$ is produced by more than one $J_p$ and $[Na^+]_i$, we would have at least two possible steady states for the time series run with that pump rate constant, and thus a poor mapping between the constant pump rate solution and the cubic pump rate model.

Indeed, the mapping between $p$ and $J_p$ is found to be bijective over the range of $J_p$s for which we are concerned by numeric methods. This proves that the analytic solution derived here is sufficient for finding a parametric solution for the cubic pump rate pump leak model used in our manuscript.
