# Supplementary material for: Biophysical models reveal the relative importance of transporter proteins and impermeant anions in chloride homeostasis
Source: eLife. 2018 Sep 27;7:e39575. doi: 10.7554/eLife.39575 (PMC6200395; doi:10.7554/eLife.39575)
Supplement: Supplementary file 2. [file elife-39575-supp2.docx]

| **Study** | **Region** | **Δ KCC2** | **Δ NKCC1** | **Δ [Cl^-^]_i_** | **Δ V_m_ (mV)** | **Δ DF (mV)** | **Weight** |
| --- | --- | --- | --- | --- | --- | --- | --- |
| Lagostena et al. (2010) | Hippocampus | ↓ 98% | ↓ 16% | ↑ 118% | -0.9 | -20.9 | 36 |
| Lee et al. (2011) | Hippocampus | ↓ 42% | ↑ 7% | ↑ 72% | 2 | -12 | 35 |
| Campbell et al. (2015) | Neocortex | ↓ 37% | ↑ 27% | ↑ 59% | 2 | -9.9 | 34 |
| Tang et al. (2015) | Spinal cord | ↓ 35% | ↓ 11% | ↑ 56% | 0.2 | -11.2 | 31 |
| MacKenzie and Maguire (2015) A | Hippocampus | ↓ 17% | No data | ↑ 37% | 1 | -7.07 | 24 |
| MacKenzie and Maquire (2015) B | Hippocampus | ↓ 4% | No data | ↑ 2% | -8 | -8.45 | 23 |
| Mahadevan et al. (2015) | Hippocampus | ↓ 20% | No data | ↑ 20% | No data | No data | 20 |
| Ferrini et al. (2013) | Spinal cord | ↓ 45% | No data | ↑ 42% | 0 | -9 | 19 |
| Coull et al. (2003) | Spinal cord | ↓ 75% | No data | ↑ 151% | -1 | -24.6 | 18 |
| **R^2^ value for regression against Δ KCC2 percentage:** | | | | 0.83 (p<0.001) | 0.045 (p<0.005) | 0.796 (p<0.001) |  |

*Table S2-1: Changes in the activity of cation chloride co-transporters are associated with changes in intracellular chloride concentration. For each study, the shift in the chloride gradient, change in KCC2 expression, and change in NKCC1 expression was extracted. NKCC1 was assumed to be unlikely to alter chloride homeostasis as adult tissue only was considered (Ben-Ari, 2002), although studies reporting non-signiﬁcant changes in NKCC1 expression were weighted higher than those that did not test NKCC1 expression. However, when studies also reported signiﬁcant expression changes in NKCC1 or functional inhibition conﬁrming that NKCC1 was contributing towards the disease-mediated shift in chloride concentration, they were excluded.*

| **Category** | | **Score** |
| --- | --- | --- |
| **Experimental preparation** | | |
| Region | *Cortical brain tissue* | 2 |
|  | *Spinal cord tissue* | 1 |
| Technique used | *Acute tissue slice* | 3 |
|  | *Organotypic tissue slice* | 2 |
|  | *Dissociated cell culture* | 1 |
| **KCC2 expression data** | | |
| Technique used to measure KCC2 expression | *RT-PCR* | 3 |
|  | *Immunoblotting* | 2 |
|  | *Immunohistochemistry* | 1 |
| Change in KCC2 expression | *Stated in-text* | 2 |
|  | *Extracted from graphic* | 1 |
| Sample size | *>20* | 4 |
|  | *10-20* | 3 |
|  | *5-9* | 2 |
|  | *<5* | 1 |
| **NKCC1 expression data** | | |
| Technique used to measure NKCC1 expression | *RT-PCR* | 3 |
|  | *Immunoblotting* | 2 |
|  | *Immunohistochemistry* | 1 |
| Change in KCC2 expression | *Stated in-text* | 10 |
|  | *Extracted from graphic* | 8 |
|  | *None* | 0 |
| Sample size | *>20* | 4 |
|  | *10-20* | 3 |
|  | *5-9* | 2 |
|  | *<5* | 1 |
| **E_GABA_ data** | | |
| Technique used to measure E_GABA_ | *Gramcidin perforated patch-clamp* | 4 |
|  | *Whole-cell patch-clamp* | 1 |
| Change in E_GABA­_ expression | *Stated in-text* | 4 |
|  | *Extracted from graphic* | 1 |
| Sample size | *>20* | 4 |
|  | *10-20* | 3 |
|  | *5-9* | 2 |
|  | *<5* | 1 |

*Table S2-2: Scoring system used to weight studies for the least squares regression meta-analysis in Figure 3C.*
